# Supplementary material for: Genome-Wide Identification of B-Box Gene Family and Candidate Light-Related Member Analysis of Tung Tree (Vernicia fordii)
Source: Int J Mol Sci. 2024 Feb 6;25(4):1977. doi: 10.3390/ijms25041977 (PMC10888079; doi:10.3390/ijms25041977)
Supplement: Supplementary file 1 [file ijms-25-01977-s001.zip › Figure S1-S4.pdf]

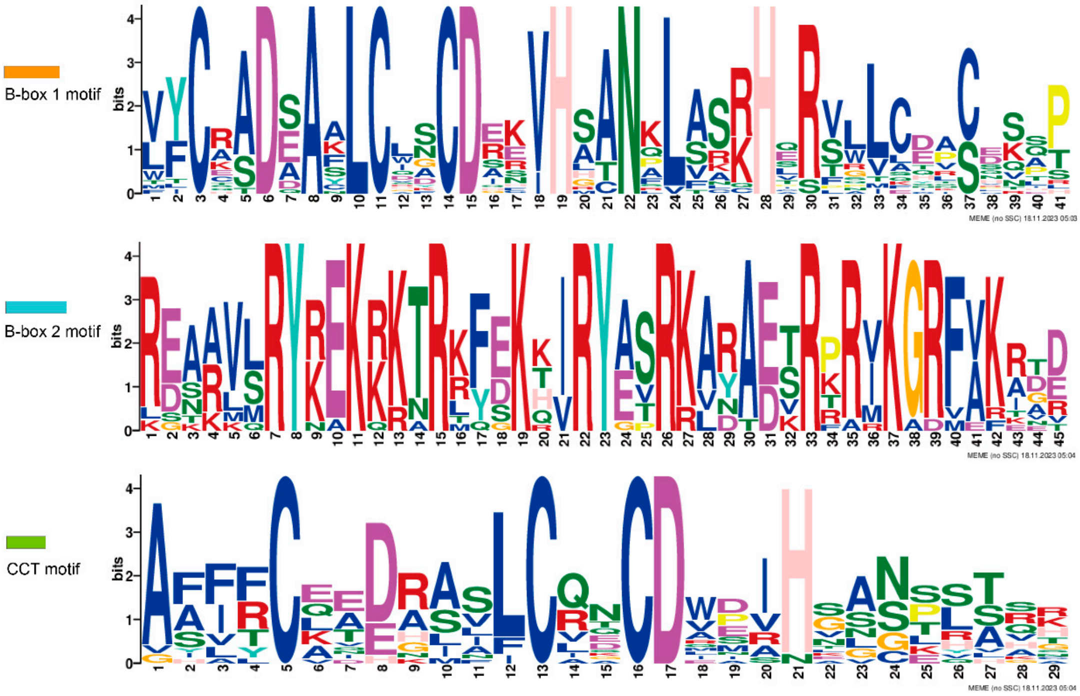

Figure S1. Typical logo of the VfBBX conserved domains.

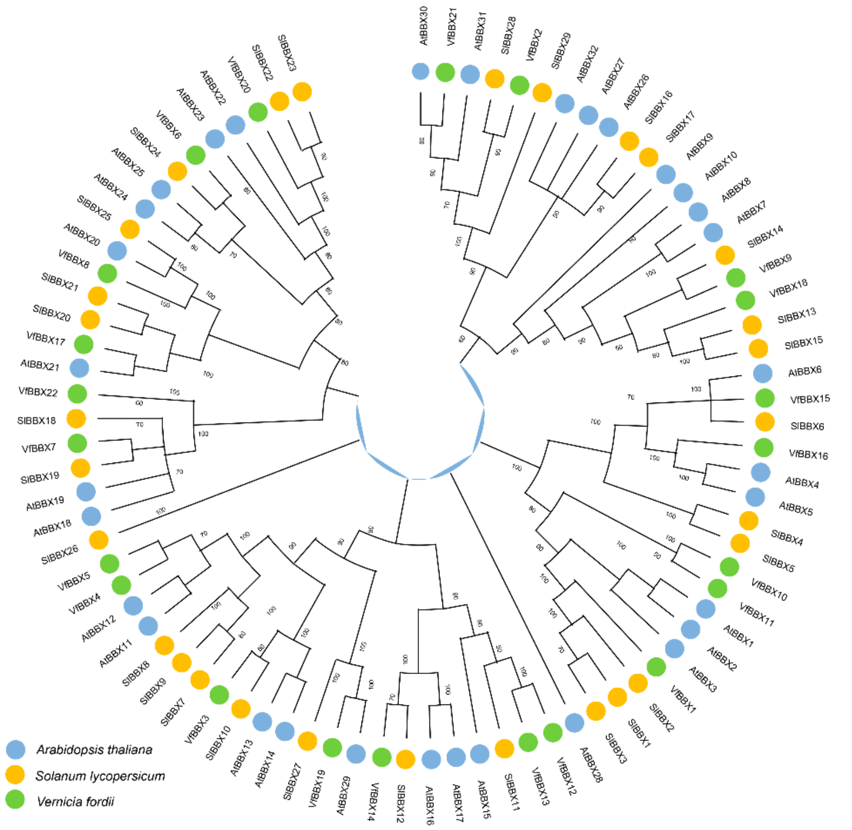

Figure S2. Phylogenetic tree of VfBBXs, SlBBXs, and AtBBXs. The unrooted phylogenetic tree was constructed by the neighbor-joining (NJ) method using 1,000 bootstrap replicates. The orange cycles represent tomato SlBBXs, blue cycles represent the AtBBXs in *Arabidopsis*, and green cycles are the VfBBXs in tung tree.

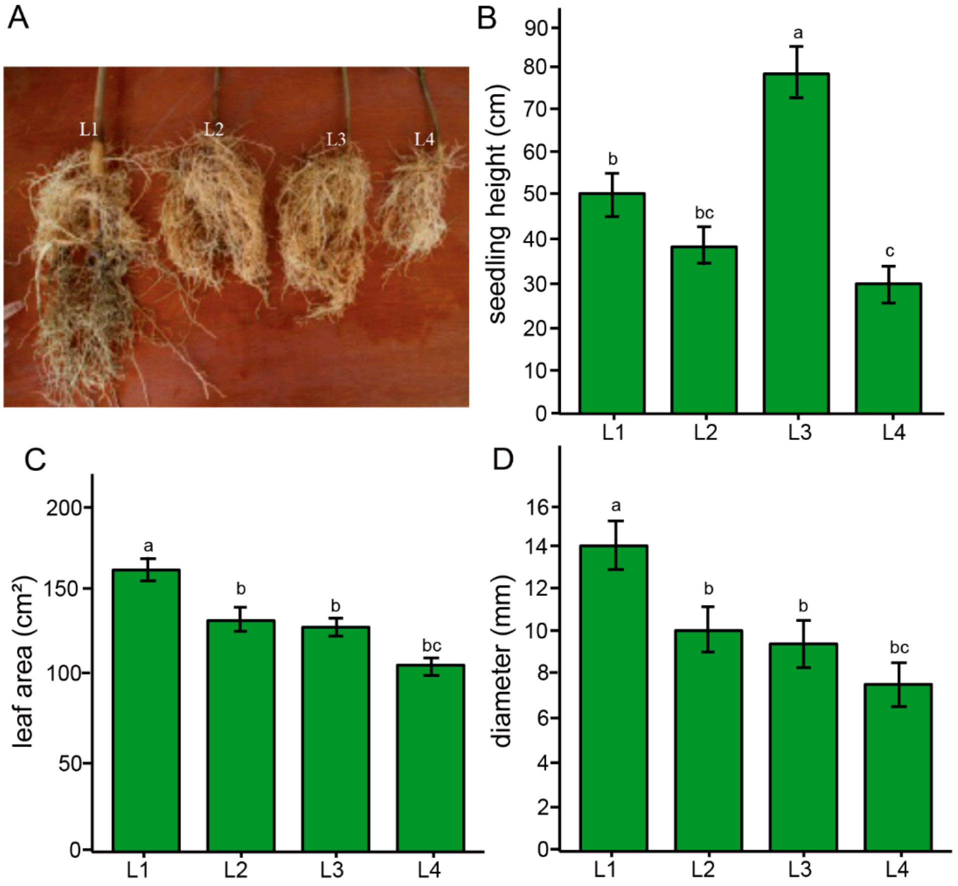

Figure S3. Physiological data of different light-shading treatments of tung tree seedlings. (A) Root phenotype of tung tree seedlings in different light-shading treatments, normal light (L1), 75% (L2), 50% (L3), and 25% (L4) light intensity levels; (B) Seedling height in different light-shading treatments; (C) Leaf area of tung tree seedlings under different light-shading treatments; (D) Diameter of tung tree seedlings under different light-shading treatments.

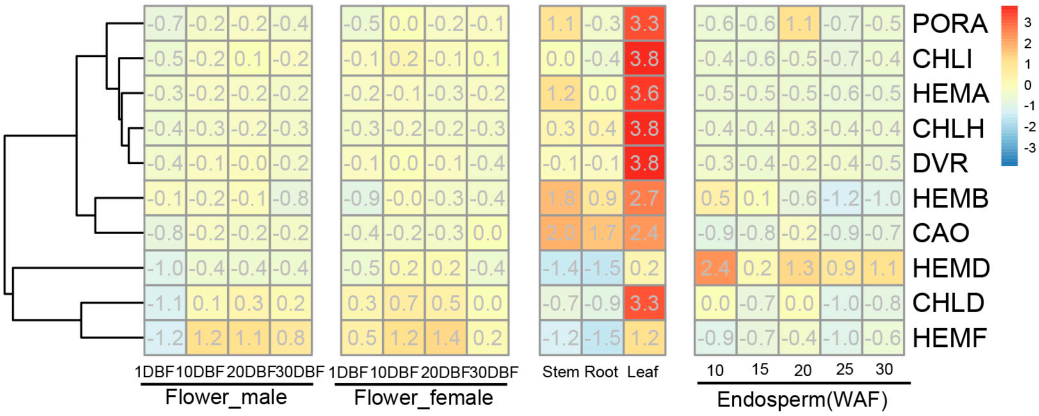

Figure S4. Expression levels of chlorophyll biosynthesis genes in different tissues of tung tree.
